# Immobilization of *Chlamydomonas reinhardtii* CLH1 on APTES-Coated Magnetic Iron Oxide Nanoparticles and Its Potential in the Production of Chlorophyll Derivatives

**DOI:** 10.3390/molecules21080972

**Published:** 2016-07-26

**Authors:** Chih-Chung Yen, Yao-Chen Chuang, Chia-Yun Ko, Long-Fang O. Chen, Sheau-Shyang Chen, Chia-Jung Lin, Yi-Li Chou, Jei-Fu Shaw

**Affiliations:** 1 Institute of Genomics and Bioinformatics, National Chung Hsing University, Taichung 40227, Taiwan; rover4rover4@gmail.com; 2 Agricultural Biotechnology Center, National Chung Hsing University, Taichung 40227, Taiwan; 3 Institute of Biomedical Engineering and Nanomedicine, National Health Research Institutes, Miaoli 35053, Taiwan; ycchuang@nhri.org.tw; 4 Institute of Plant and Microbial Biology, Academia Sinica, Taipei 11529, Taiwan; g874222@gate.sinica.edu.tw (C.-Y.K.); ochenlf@gate.sinica.edu.tw (L.-F.O.C.); 5 Department of Biological Science & Technology, I-Shou University, Kaohsiung 840, Taiwan; cd710402@hotmail.com (S.-S.C.); didi0212.bce98g@g2.nctu.edu.tw (C.-J.L.)

**Keywords:** *Chlamydomonas reinhardtii* chlorophyllase 1, immobilized enzyme, magnetic iron oxide nanoparticles, chlorophyll derivatives

## Abstract

Recombinant *Chlamydomonas reinhardtii* chlorophyllase 1 (CrCLH1) that could catalyze chlorophyll hydrolysis to chlorophyllide and phytol in vitro was successfully expressed in *Escherichia coli*. The recombinant CrCLH1 was immobilized through covalent binding with a cubic (3-aminopropyl) triethoxysilane (APTES) coating on magnetic iron oxide nanoparticles (MIONPs), which led to markedly improved enzyme performance and decreased biocatalyst costs for potential industrial application. The immobilized enzyme exhibited a high immobilization yield (98.99 ± 0.91 mg/g of gel) and a chlorophyllase assay confirmed that the immobilized recombinant CrCLH1 retained enzymatic activity (722.3 ± 50.3 U/g of gel). Biochemical analysis of the immobilized enzyme, compared with the free enzyme, showed higher optimal pH and pH stability for chlorophyll-a hydrolysis in an acidic environment (pH 3–5). In addition, compared with the free enzyme, the immobilized enzyme showed higher activity in chlorophyll-a hydrolysis in a high temperature environment (50–60 °C). Moreover, the immobilized enzyme retained a residual activity of more than 64% of its initial enzyme activity after 14 cycles in a repeated-batch operation. Therefore, APTES-coated MIONP-immobilized recombinant CrCLH1 can be repeatedly used to lower costs and is potentially useful for the industrial production of chlorophyll derivatives.

## 1. Introduction

Chlorophyllase (Chlase, EC 3.1.1.14) is a crucial enzyme involved in chlorophyll (Chl) degradation pathways during leaf senescence, fruit ripening, and pathogen infection [1,2,3]. Chl dephytylation can be divided into two pathways: First, Chlase catalyzes thylakoid anchoring phytol chain hydrolysis to yield chlorophyllide (Chlide) as an intermediate that occurs before magnesium (Mg) removal. Second, Mg dechelation occurs before dephytylation with Mg-free Chl (pheophytin) as an intermediate; phytol is then removed by pheophytinase [2]. Chlase exists in many species that contain Chl, such as higher plants [4,5,6,7,8,9,10,11,12], algae [13,14,15,16,17,18], and cyanobacteria [19], from which enzymes have been isolated. All Chlase proteins have a highly conserved lipase motif (GXSXG) and a catalytic triad (Ser-His-Asp), in which the serine acts as a nucleophile in the hydrolysis reaction [1,4,7].

**Figure 1:**
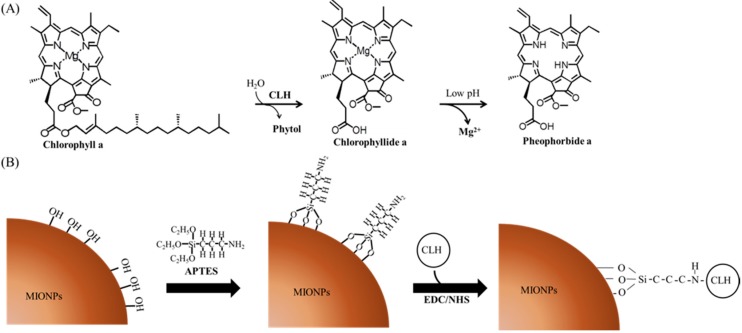
Schematic of enzyme reaction and immobilization. (**A**) Recombinant CrCLH1 catalyzed Chl-a to Chlide-a. The Mg ion bond with Chlide-a was unstable under low pH conditions and was followed by Mg dechelation (**B**) Reaction of the (3-aminopropyl) triethoxysilane (APTES) coating with magnetite nanoparticles used to immobilize recombinant CrCLH1 on the magnetic iron oxide nanoparticles (MIONPs). *N*-ethyl-*N*-3-dimethylaminopropyl carbodiimide (EDC) and *N*-hydroxysuccinimide (NHS) were used to form activating carboxyl groups for reaction with other amine-containing molecules.

Previously, Chlase was used to remove Chl residues to increase the oxidative stability of canola oil [20]. Recently, several studies have indicated that Chl derivatives such as Chlide, pheophorbide, and phytol possess anticarcinogenic, antioxidant, antiviral, and antiinflammatory properties [21,22,23,24,25]. Chl derivatives can be nontoxic, easily extracted, cost-effective, and environmentally friendly organic agents against *Anopheles gambiae* larvae because of their photodynamic toxicity [26]. Our previous study successfully isolated the *Chlase1* gene from *Chlamydomonas reinhardtii* and expressed recombinant *C. reinhardtii* Chlase 1 (CrCLH1) in the *Escherichia coli* expression system for biochemical analysis. Furthermore, enzyme-kinetic data revealed that the recombinant CrCLH1 had higher catalytic efficiency (kcat/Km) toward Chl-a than Chl-b or bacteriochlorophyll-a [18]. In addition, the Mg ion bond with Chl was unstable under low pH conditions and was followed by Mg dechelation. Thus, recombinant CrCLH1 catalyzed Chl-a to pheophorbide-a under acidic conditions (pH 5) in one step (Figure 1A). Therefore, recombinant CrCLH1 can be used as a biocatalyst to produce Chlide derivatives that have potential as drugs for the biotechnology and pharmaceutical industries.

Free enzymes are difficult to recycle and reuse and are highly susceptible to inactivation under external conditions. During enzyme immobilization, the maintenance of enzyme stability during any biochemical reaction is highly challenging. Enzyme immobilization techniques can be categorized into adsorption, entrapment, affinity, and covalent binding [27]. Traditional support materials used for enzyme immobilization can be divided into natural polymers (such as alginate, chitosan, collagen, carrageenan, gelatin, cellulose, starch, pectin, and Sepharose), ion exchange resins (such as Amberlite and DEAE cellulose), and inorganic materials (such as zeolites, ceramics, Celite, silica, glass, modified carbon, and charcoal) [27]. Recently, the importance of the development of materials synthesis with nanoscale precision is well realized. Nanotechnology is a technology to fabricate nanostructured materials, including magnetic nanoparticles are a novel material that can be used in enzyme immobilization. Previous studies, the application of magnetic nanoparticles were an emerging nanomaterial for enzyme immobilization [28,29,30,31,32]. In the present study, magnetic iron oxide nanoparticles (MIONPs) were used for support in all procedures. MIONPs can be easy collected or guided using an external magnetic field that can be used in enzyme immobilization. Therefore, MIONPs are widely used in enzyme recycling and reuse. In our previous studies, we entrapped BoCLH1 in MIONP-loaded alginate-composite beads. The immobilized BoCLH1 in MIONP-loaded alginate composite beads was collected using a magnet and the enzyme catalytic effect was maintained for only six cycles. However, the stability of BoCLH1 in MIONP-loaded alginate composite beads did not increase significantly under low pH conditions [32]. To select an appropriate enzyme immobilization strategy for improving Chlase pH stability and recycling, the covalent attachment was used in the present study. Covalent attachment is very likely the most effective strategy for increasing enzyme stability [33,34]. Our strategy involved preparing MIONPs coated with (3-aminopropyl) triethoxysilane (APTES), which possesses an active group of NH_2_ was conjugated to recombinant CrCLH1 by covalent band [35]. Figure 1B shows that APTES-coated MIONPs can be reacted with *N*-ethyl-*N*-3-dimethylaminopropyl carbodiimide (EDC) and *N*-hydroxysuccinimide (NHS), which are water-soluble carbodiimide chemistry reagents that provide active carboxyl groups for reaction with other amine-containing molecules [36]. Initially, EDC forms an amine-reactive O-acylisourea intermediate through the reaction with the carboxyl group of the APTES-coated MIONPs [35]. It then reacts with the amine group of the recombinant CrCLH1 surface to produce a stable isopeptide bond. Therefore, a simple immobilization support of APTES-coated MIONPs was used in this study.

Agricultural waste usually contains large amounts of Chl that are only used for natural degradation. An opportunity exists to increase the value of these wastes and provide greater contributions to biotech medicine by converting Chl into high-value nutraceuticals for human health or pharmaceutical products. Enzymes are highly active, selective and specific tools for performing complex chemical processes under mild physiological conditions. However, once immobilized on suitable materials, enzymes can be used as reactors for chemical transformations even under harsh conditions. Thus, the integration of enzyme engineering with material science is a very exciting goal. APTES-coated MIONPs immobilization of recombinant CrCLH1 can increase the enzyme stability or improved environmental tolerance and increased enzyme recycling and reuse. Additionally, it represents a potential catalytic reactor for the industrial production of Chl derivatives for biomedical applications. In order to design and synthesize more sophisticated materials and structures currently the central concept is changing from nanotechnology to nanoarchitectonics [37,38,39]. Nanoarchitectonics is a novel concept involving the design and synthesis of more sophisticated materials and structures for environmental, biological and medical applications [37,38,39,40].Recent developments in enzyme immobilization for advanced technology include the application of nanoarchitectonics [41]. We hope that this research will be a further contribution and participation in nanoarchitectonics.

## 2. Results and Discussion

### 2.1. Synthesis of MIONPs and APTES-Coated MIONPs

**Figure 2:**
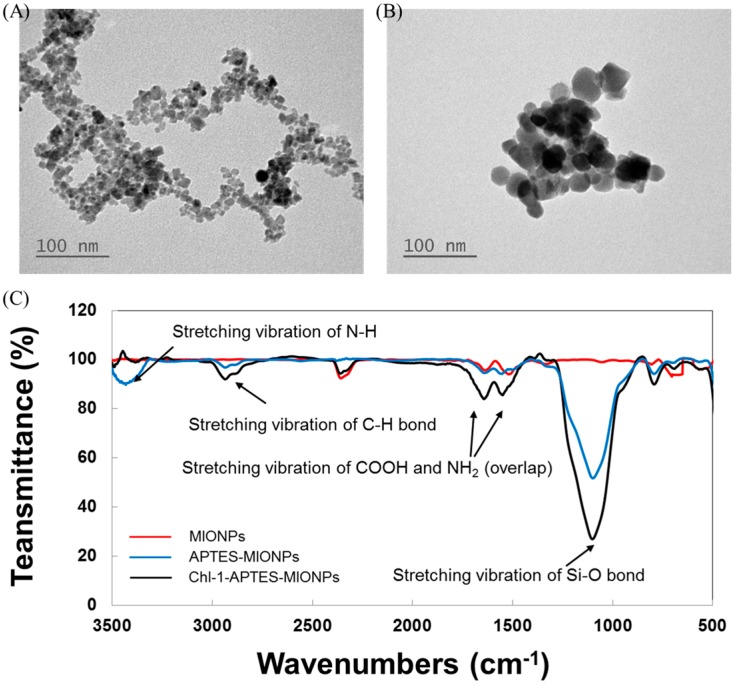
Synthesis of MIONPs and APTES-coated MIONPs. (**A**) TEM micrograph: pure MIONPs and (**B**) APTES-coated MIONPs; (**C**) FTIR analysis of pure MIONPs (red line) and APTES-coated MIONPs (blue line).

Figure 2A shows a transmission electron microscopy (TEM) image of the MIONPs, which indicates that most of the particles are quasispherical and have an average diameter of 7.5 ± 2.3 nm. The TEM image in Figure 2B depicts APTES-coated MIONPs with an average diameter of 10.2 ± 3.4 nm. To investigate the composition of the MIONPs, we performed scanning electron microscopy-energy dispersive X-ray spectroscopy (EDX) elemental analysis on several samples. An EDX spectrum for the produced APTES-coated MIONPs is illustrated in Figure S1. The EDX results indicate that the major component of the synthesized nanoparticles (approximately 95%) is Fe, and that the Fe:Si composition is similar to the 96.75% Fe: 3.25% Si composition reported for nanoparticles synthesized by Ma et al. [35]. Compared with the Fourier transform infrared spectroscopy (FTIR) spectra of unmodified MIONPs (as shown in Figure 2C), the APTES-coated MIONPs and CrCLH1-APTES-MIONPs possess absorption bands at 2870 and 2920 cm^−1^ due to the stretching vibrations of the C-H and C=O bonds. The broad band at approximately 1000 cm^−1^ and medium strength bands at approximately 1300–1600 cm^−1^ arise from stretching vibrations of the Si-O bonds and C-N bonds, respectively. Because these absorption bands can be assigned to the C-N stretch and NH_2_ vibration of APTES, the OH groups on MIONOs were evidently efficiently replaced by APTES. All of these findings evidence the presence of APTES and CrCLH1 on MIONPs.

### 2.2. Immobilization of Recombinant CrCLH1

**Figure 3:**
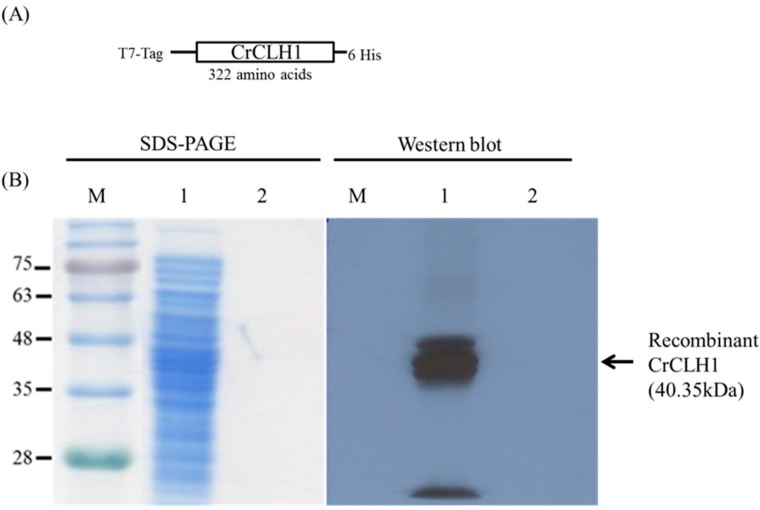
Capacity of APTES-coated MIONPs to immobilize the crude cell lysate with recombinant CrCLH1. (**A**) Schematic representation of the recombinant CrCLH1; (**B**) SDS-PAGE and western blot analysis of the crude cell lysate before (Lane 1) and after (Lane 2) enzyme immobilization. Western blot analysis was performed using a monoclonal anti-His tag antibody. M: marker; Lane 1: crude cell lysate; Lane 2: crude cell lysate after binding. The arrow indicates the location of recombinant CrCLH1. The molecular mass of the recombinant CrCLH1 was 40.35 kDa. The protein gel was stained with Coomassie blue.

To increase the potential for industrial application of CrCLH1, it was immobilized to reducing cost and increase its stability. Recombinant CrCLH1 was immobilized on MIONPs with a covalent bond by using the method described in [36]. The deduced *CrCLH1* gene encoded 322 amino acids. The recombinant CrCLH1 fused with an N-terminal T7-Tag, and the C-terminal histidine tag (His tag) (Figure 3A) was overexpressed in the *E. coli* BL21 (DE3) strain.

The cells were lysed using a sonicator and the supernatant harvested for enzyme immobilization. The APTES-coated MIONPs were employed as support for immobilization through covalent binding with recombinant CrCLH1. The enzyme immobilization procedure is shown in Figure 1B. To confirm the capacity of APTES-coated MIONPs to immobilize recombinant CrCLH1, covalent binding of the recombinant CrCLH1 to the APTES-coated MIONPs was performed (Lane 1, Figure 3B); the unbound proteins remained in the spent crude cell lysate after enzyme immobilization (Lane 2). This result indicates that the crude cell lysate was successfully immobilized on APTES-coated MIONPs and that amount of unbound proteins remaining in the spent crude cell lysate after immobilization was negligible. Western blot analysis was performed using a monoclonal anti-His tag antibody. The western blot analysis results also reveal that the recombinant CrCLH1 was successfully immobilized on APTES-coated MIONPs. The immobilized enzyme was used for determining temperature influences and biochemical properties such as pH.

### 2.3. Immobilization Yield and Enzyme Capacity

To investigate the effects of the APTES-coated MIONPs content on the efficiency of enzyme immobilization, we used 0.1 g dry weight of the APTES-coated MIONPs incubated with 2.76 mg/mL crude cell lysate containing the recombinant CrCLH1 as a support and calculated the enzyme capacity (mg/g of gel) and immobilization yield (%) through a Bradford dye-binding assay. Table 1 shows the amount of protein binding and the protein immobilization yield of the support. The APTES-coated MIONPs exhibited a protein binding value of 32.1 ± 0.3 mg/g of gel and had a superior immobilization yield of 98.99% ± 0.91%. These results indicated that the immobilization yield of the APTES-coated MIONPs was higher than that of only MIONP entrapment or IMAC desorption with the BoCLH1 enzyme (62%, or 74.3% ± 6.5%) in our previous studies [32,42], because the covalent binding interaction was strong between the enzyme and the surface of the MIONPs [43], resulting in a maximum load. Overall, APTES-coated MIONPs are considered a favorable support for enzyme immobilization because of their maximum surface area per unit mass, high enzyme loading capability, and minimal diffusional limitations [44].

The enzyme activity of the immobilized CrCLH1 was 722.3 ± 50.3 U/g of gel, and the specific activity of the immobilized enzyme was 131.0 ± 9.1 U/mg of protein. The specific activity of the non-immobilized enzyme was 186 ± 22 U/mg of protein, which is higher than that of the immobilized enzyme. This result might be attributed to the strong interaction limiting enzyme movement, resulting in decreased enzyme activity [45]. The specific activity of the non-immobilized enzyme in the spent cell lysate was only 4.1 ± 0.3 U/mg of protein. This does not illustrate that the covalent bonding of the APTES-coated MIONPs with the recombinant CrCLH1 depends on the purity level; however, the target protein may be immobilized faster than certain contaminant proteins [43]. Moreover, this result illustrated that the immobilized enzyme could be collected very rapidly, easily, and efficiently (Figure S2).

### 2.4. Effects of Optimal pH and pH Stability of the Free and Immobilized Enzymes

**Figure 4:**
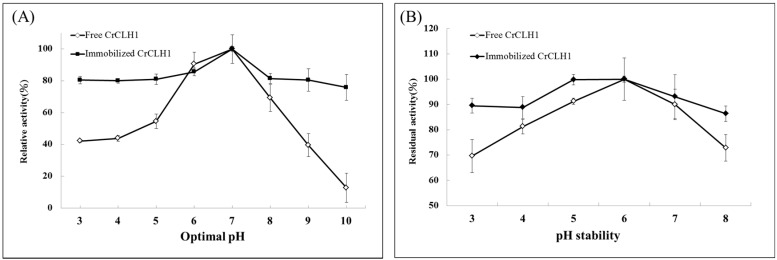
Effects of optimal pH and pH stability of the free enzyme (◊) and the immobilized enzyme (♦). (**A**) Activity analyses of the free and immobilized enzymes were conducted at 30 °C at pH values of 3–10 for 1 min; (**B**) The free and immobilized enzymes were in Cubated at different pH values ranging from pH 3 to 8 for 10 min prior to activity analysis at 30 °C. Data represent means ± the standard deviation of three independent experiments.

The effects of optimal pH and pH stability on the activity of the free (crude cell lysate) and immobilized enzymes (crude cell lysate bound to APTES-coated MIONPs) were investigated. The Chlase activity of the free and immobilized enzyme preparations was measured at 30 °C at pH values ranging from 3 to 10. Analysis of the optimal pH (Figure 4A) for the free and immobilized enzymes resulted in pH 7. Hence, we defined pH 7 as 100%. The free enzyme retained >90% of its maximal activity pH 6 [18]. The free enzyme activity declined significantly at pH 5, and the enzyme retained approximately 42% and 44% of its specific activity at pH 3 and 4, respectively; however, the relative activity of the free enzyme declined significantly at pH 8–10. Similar to the free enzyme, the immobilized enzyme exhibited an optimal reaction at pH 7 and retained 86% of its relative activity at pH 6. In addition, the immobilized enzyme retained >76% of its activity at high pH values (pH 8–10). Similar observations have been reported by other researchers [31,36]. These reports confirm that covalently immobilized enzymes are much more stable than free enzymes at high pH values. The immobilized enzyme retained a higher relative activity than did the free enzyme in an acidic (pH 3–5) environment. Previous studies have indicated that the immobilization of enzymes onto MIONPs improved low pH tolerance [28,31]. Therefore, the covalent bonding of recombinant CrCLH1 to APTES-coated MIONPs promote a rigidification of the protein structure and reduce any conformational change of the immobilized enzyme under low pH condition [33].

The stability of pH on the free and immobilized enzymes was determined by measuring their Chl-a hydrolysis activity. As shown in Figure 4B, the free enzyme had the highest relative activity (100%) in hydrolyzing Chl-a at pH 6; however, the relative activity of the free enzyme sharply decreased after incubation at a lower pH (3 to 5) and higher pH (7 to 8) for 10 min; all enzymes lost approximately 10% to 30% of activity. Figure 4B illustrates that the stability of the immobilized enzyme was the highest at pH 6. Furthermore, the pH stability of the immobilized enzyme was broader and all activity values were >85% of the maximum (100% at pH 6) in the 3–8 pH range. The immobilized enzyme was more acid-resistant because relative activity of approximately 99.7% was observed at pH 5, and broad pH stability was observed between pH 3 and 4. This result indicates that the immobilized enzyme exhibits higher tolerance under acidic conditions than does the free enzyme because of the strong interaction between proteins and APTES-coated MIONPs. Thus, the covalent bonding of recombinant CrCLH1 to APTES-coated MIONPs is crucial to the success of potential biological catalysts for the conversion of Chl-a to pheophorbide-a under acidic conditions in one step, although the activity is gradually reduced compared with that of the free enzyme.

### 2.5. Optimal Temperature and Thermostability of Free and Immobilized Enzymes

**Figure 5:**
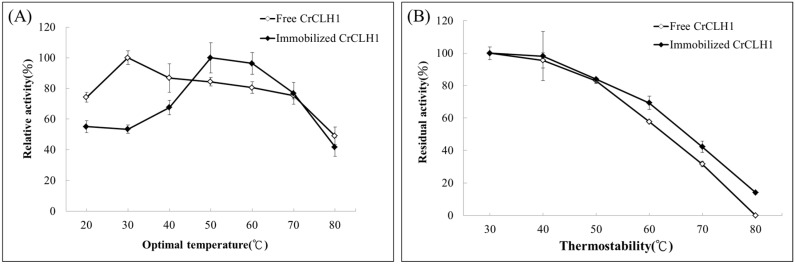
Optimal temperature and thermostability of the free enzyme (◊) and the immobilized enzyme (♦). (**A**) The free and immobilized enzymes were incubated at different temperatures (30 °C–80 °C) for 10 min prior to activity analysis at pH 8. Specific activity was measured according to the standard Chlase assay; (**B**) Activity analyses of the free and immobilized enzymes were conducted at different temperatures (20 °C–80 °C) for 1 min. Data represent means ± the standard deviation of three independent experiments.

Chlase activity assays of free and immobilized enzyme preparations were performed at pH 7.4 at various temperatures, ranging from 20 °C to 80 °C. The optimal temperature (Figure 5A) of the free enzyme was 30 °C, and we defined 30 °C as 100%. The relative activity of the free enzyme remained at 80%–86% at 40 °C to 60 °C. Moreover, the relative activity of the free enzyme decreased to 76% at 70 °C and sharply decreased to 49% when the temperature was above 80 °C. By contrast, the relative activity of the immobilized enzyme was lower (53%–68%) than that of the free enzyme (74%–100%) at 20 °C–40 °C. However, the optimal temperature of the immobilized enzyme shifted to 50 °C; it retained 96% of the maximum activity at 60 °C. Compared with the free enzyme, the immobilized enzyme exhibited higher relative activity at higher temperatures such as 50 °C and 60 °C. This result indicated that the immobilized enzyme likely required a higher activation energy for reorganizing the structure appropriate for substrate binding [46]. However, at temperatures of 70 °C and 80 °C, the relative activity curves of the free and immobilized enzymes were similar to those at the optimal temperature. These results indicate that enzyme immobilization methods might exert a protective effect on enzymes at high temperatures and enable them to tolerate high temperatures for industrial applications.

Figure 5B shows that regarding thermostability, the free and immobilized enzymes both have higher activity at 40 °C. At 60 °C, a relatively higher activity (69%) was observed for the immobilized enzyme compared with that of 58% for the free enzyme. The immobilized enzyme retained 42% relative activity at 70 °C, compared with that of 32% for the free enzyme. Thus, the covalent bond between the APTES-coated MIONPs and immobilized enzymes may remain unaltered during any conformational change induced by a distorting agent, such as heat and extreme pH values [28,31]. Accordingly, applying the APTES-coated MIONPs is an effective immobilization method for reducing any conformational change involved in enzyme inactivation and effectively increases the enzyme stability.

### 2.6. Operational Stability

**Figure 6:**
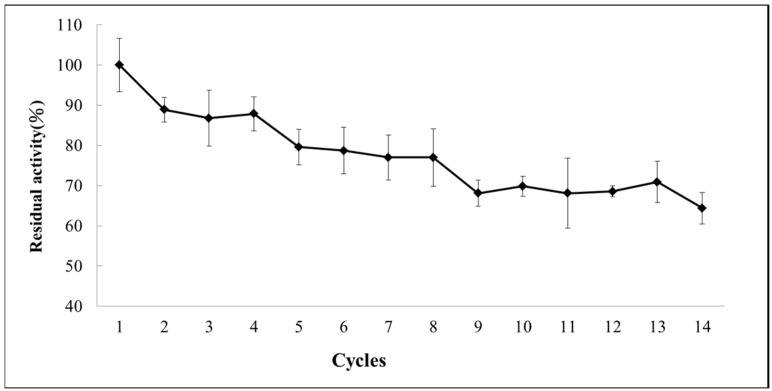
Reusability of the immobilized enzyme. The residual activity of the immobilized enzyme (♦) was monitored at room temperature in a reaction buffer containing 100 μM Chl-a for 10 min.

Immobilized enzymes that can be easily recycled are advantageous for reducing industrial costs. The operational stability of the immobilized recombinant CrCLH1 was determined by monitoring the residual activity of the immobilized enzyme after recycling at room temperature and pH 7.4 for 30 min. We defined the residual activity in the first analysis as 100%. The immobilized recombinant CrCLH1 was used 14 times for the catalytic reaction. The immobilized recombinant CrCLH1 on APTES-coated MIONPs was collected with an external magnet (Figure S2). Each reaction lasted 30 min. The reaction medium was replaced with a fresh reaction buffer containing Chl-a. As shown in Figure 6, the immobilized recombinant CrCLH1 retained 87%–89% of the residual activity after the second to fourth cycles compared with the first cycle. The residual activity of the immobilized recombinant CrCLH1 was 77%–80% after the fifth to eighth cycles. However, the activity of the immobilized recombinant CrCLH1 was further reduced (68%–71%) after the 9th–13th cycles. After the 14th cycle, the residual activity sharply decreased to 64%. These results clearly evidence that immobilizing recombinant CrCLH1 on APTES-coated MIONPs is an effective method for recycling the enzyme more than 14 times and can reduce biocatalyst costs for industrial applications.

## 3. Materials and Methods

### 3.1. Materials

NHS, EDC, and APTES were acquired from Sigma (St. Louis, MO, USA).

### 3.2. Synthesis of MIONPs

Magnetite was produced according to the method of Kang [47]. In brief, FeCl_3_ (5.2 g) and FeCl_2_ (2.0 g) were successively dissolved in purified, deoxygenated water (25 mL, containing 0.85 mL of 12.1 N HCl) through stirring. The solution was then added dropwise into 1.5 M NaOH solution (250 mL) under vigorous stirring, which generated an instant black precipitate (Fe_3_O_4_). The obtained Fe_3_O_4_ was washed immediately with water to remove unreacted chemicals by magnetic decantation using an external magnet. Purified deoxygenated water was added to the precipitate and the solution was decanted after centrifugation at 4000 rpm. After repeating the last procedure three times, 0.01 M HCl solution (500 mL) was added to the precipitate (with stirring) to neutralize the anionic charges of the nanoparticles.

### 3.3. APTES-Coated MIONPs

Twenty five milliliters of the magnetite colloid solution prepared as described was diluted to 150 mL with ethanol containing 0.01% (*v*/*v*) acetic acid. The solution was then treated using ultrasonic waves for 30 min. Subsequently, APTES (NH_2_(CH_2_)_3_Si(OC_2_H_5_)_3_, 35 mL) was added while rapidly stirring the solution for 7 h. The resultant solution was washed with ethanol five times and then dried into powder at room temperature under vacuum.

### 3.4. Characterization of MIONPs and APTES-Coated MIONPs

The particle size and morphology of the samples were determined through TEM (JEM-2000 EX II, JEOL, Tokyo, Japan) at a voltage of 80 kV. The compositions of Fe_3_O_4_ and APTES-Fe_3_O_4_ were obtained through EDX (S-3000N, Hitachi, Tokyo, Japan). FTIR spectroscopy (Nicolet 750, Thermo Nicolet Corp., Madison, WI, USA)) of the samples was employed to examine the chemical bonds between Fe_3_O_4_ and APTES.

### 3.5. Synthesis of Recombinant CrCLH1-APTES-Coated MIONPs

Covalent binding of recombinant CrCLH1 to the MNPs-NH_2_ was performed using a modification of the EDC/NHS reaction. MNPs-NH2 (0.1 g) was treated with 0.05 M Tris-HCL (300 μL, containing 1 mg of NHS and 1.6 mg of EDC, pH 8). Carboxyl groups of recombinant CrCLH1 were activated using an EDC/NHS solution for 30 min. Following activation, crude cell lysate (700 μL) was added to form a mixed solution and allowed to react at room temperature for 12 h. The resultants were washed using distilled water alternatively for removing unreacted chemicals by magnetic decantation using an external magnet.

### 3.6. Preparation of Enzymes

We constructed recombinant CrCLH1 in a pET-21a (+) expression vector in a previous study [18]. *E. coli* cells harboring plasmid pET-21a-CrCLH1 were grown in 1 L of Luria-Bertani medium (USB Corp., Cleveland, OH, USA) containing 100 g/mL ampicillin at 37 °C for 16 h. Protein overexpression was induced by adding a final concentration of 0.1 mM IPTG to a refreshed culture at 16 °C for more than 20 h. After centrifugation (13,000 rpm for 10 min at 4 °C), the harvested cell pellet was suspended and lysed using a French press (Thermo Scientific, Waltham, MA, USA)) in a TE buffer (100 mM Tris·HCl, 1 mM EDTA, pH 8). The recombinant CrCLH1 concentration was quantified using the Bradford assay (Bio-Rad, Hercules, CA, USA). The protein concentration of the crude cell lysate was approximately 2.76 mg/mL.

### 3.7. Chlase Assay of the Immobilized Enzyme

The Chlase activity assay was measured according to the procedure recommended by Chou et al. [42], which involved mixing the immobilized CrCLH1 (0.05 g), a reaction buffer (500 μL, 100 mM sodium phosphate, pH 7.4, and 0.24% Triton X-100), and ethanol-dissolved Chl-a (150 μL, from *Anacystis nidulans* algae, Sigma). The reaction mixture was incubated in a shaking water bath at 40 °C. The amount of product formed had a linear relationship with reaction time within 1 min. Therefore, in the following assay, we performed the reaction for 1 min at 40 °C to measure the initial velocity. The free enzyme assay reaction mixture contained CrCLH1 (100 μL), a reaction buffer (500 μL, 100 mM sodium phosphate, pH 7, and 0.24% Triton X-100) and ethanol-dissolved Chl-a (150 μL, 1 mM). The enzyme reaction was stopped by transferring the reaction mixture to a new centrifuge tube containing 1 mL of a stop reaction buffer (ethanol/hexane/10 mM KOH = 4:6:1 (*v*/*v*)). The mixture was vortexed and centrifuged at 13,000 rpm for 3 min to induce phase separation. The aqueous ethanol layer was then collected (Chlide-a remained). The absorbance of the aqueous ethanol phase was measured at 667 nm for Chlide-a by using a spectrophotometer. The amount of each product in the ethanol layer was estimated from the millimolar extinction coefficient of 81 mM^−1^·cm^−1^ for Chlide-a.

### 3.8. Biochemical Analyses of the Free and Immobilized Enzymes

To determine their optimal pH values, the free and immobilized enzymes were each examined in a 50 mM sodium acetate buffer with a pH range of 3–5 and in a Good’s buffer (50 mM each of Bicine, CAPS, and bis-Tris propane) within pH range of 6–10; the buffers contained Chl-a. The pH stability assay was performed with the free and immobilized enzymes, which were preincubated with a Good’s buffer solution containing 0.2% Triton X-100 at different pH values (pH 3–8) for 10 min at 30 °C. The treated free and immobilized enzymes were subsequently analyzed in a pH 7.4 reaction buffer with Chl-a at 30 °C for 1 min.

To determine the optimal temperature, the free and immobilized enzymes were each incubated in a reaction buffer with Chl-a in a temperature range of 30 °C–80 °C for 1 min. A subsequent thermal stability assay was performed to incubate the free and immobilized enzymes under temperatures ranging from 30 °C to 80 °C for 10 min, and the enzymes were subsequently cooled prior to activity analysis. The treated free and immobilized enzymes were then analyzed in a reaction buffer (pH 7.4) with Chl-a at 30 °C for 1 min. 

### 3.9. Operational Stability

The operational stability of the immobilized recombinant CrCLH1 was determined by monitoring the residual activity of the immobilized enzyme after each cycle. The reaction was performed in a reaction buffer (pH 7.4) at room temperature for 10 min. After each cycle, the immobilized enzyme was recovered using a magnet.

## 4. Conclusions

Chlase is considered a potential catalytic reactor for the industrial production of Chl derivatives. In this study, we successfully immobilized recombinant CrCLH1 on APTES-coated MIONPs through covalent binding. The covalent binding technique exhibited a higher degree of protein binding (32.06 ± 0.3 mg/g of gel) and higher immobilization yields (98.99% ± 0.91%) compared with those in our previous studies. Moreover, immobilized recombinant CrCLH1 showed higher enzymatic activity (722.3 ± 50.3 U/g of gel) as well as specific activity (131.0 ± 9.13 U/mg of protein) compared with recombinant BoCLH1 immobilized in MIONP-loaded alginate composite beads [32] or in DIAION^®^CR11-Cu(II) [42]. The pH effect assay indicated that the activity of the immobilized enzyme was increased at low pH (pH 3–5) compared with that of the free enzyme. Compared with the free enzyme, the immobilized enzyme exhibited slightly higher activity in a high temperature environment (50 °C–60 °C). The immobilized CrCLH1 retained approximately 64% of its residual activity after 14 cycles. On the basis of these results, recombinant CrCLH1 binding to APTES-coated MIONPs is an effective technique for reducing costs and improving enzyme stability in industrial applications.

## Figures and Tables

**Table 1:** Characterization of the free and immobilized CrCLH1.

Samples	Amount of Protein Binding (mg/g Gel)	Immobilization Yield (% ^b^)	Enzymatic Activity (U ^c^/g Gel)	Specific Activity of Immobilized Enzymes (U/mg Protein)	Specific Activity of Non-Immobilized Enzymes (U/mg Protein)
Free CrCLH1	0	ND ^a^	ND	ND	186 ± 22
Immobilized CrCLH1	32.1 ± 0.3	98.99 ± 0.91	722.3 ± 50.3	131.0 ± 9.1	4.1 ± 0.3

^a^ Not detected; ^b^ Immobilization yield (%) was defined as follows: Immobilization yield (%) = [Total enzyme − nonimmobilized enzyme (mg)/Total enzyme (mg)] × 100; ^c^ The enzyme activity was determined by measuring the absorbance of the aqueous EtOH phase with Chlide-a at 667 nm. One unit of Chlase activity is defined as described in the Experimental Section.

## Supplementary Materials

Supplementary materials can be accessed at: http://www.mdpi.com/1420-3049/21/8/972/s1.
