# Supplementary material for: Immobilization of Chlamydomonas reinhardtii CLH1 on APTES-Coated Magnetic Iron Oxide Nanoparticles and Its Potential in the Production of Chlorophyll Derivatives
Source: Molecules. 2016 Jul 26;21(8):972. doi: 10.3390/molecules21080972 (PMC6273557; doi:10.3390/molecules21080972)
Supplement: Supplementary file 1 [file molecules-21-00972-s001.pdf]

Supplementary Materials: Immobilization of *Chlamydomonas reinhardtii* CLH1 on APTES-Coated Magnetic Iron Oxide Nanoparticles and Its Potential in the Production of Chlorophyll Derivatives

Chih-Chung Yen, Yao-Chen Chuang, Chia-Yun Ko, Long-Fang O. Chen, Sheau-Shyang Chen, Chia-Jung Lin, Yi-Li Chou and Jei-Fu Shaw

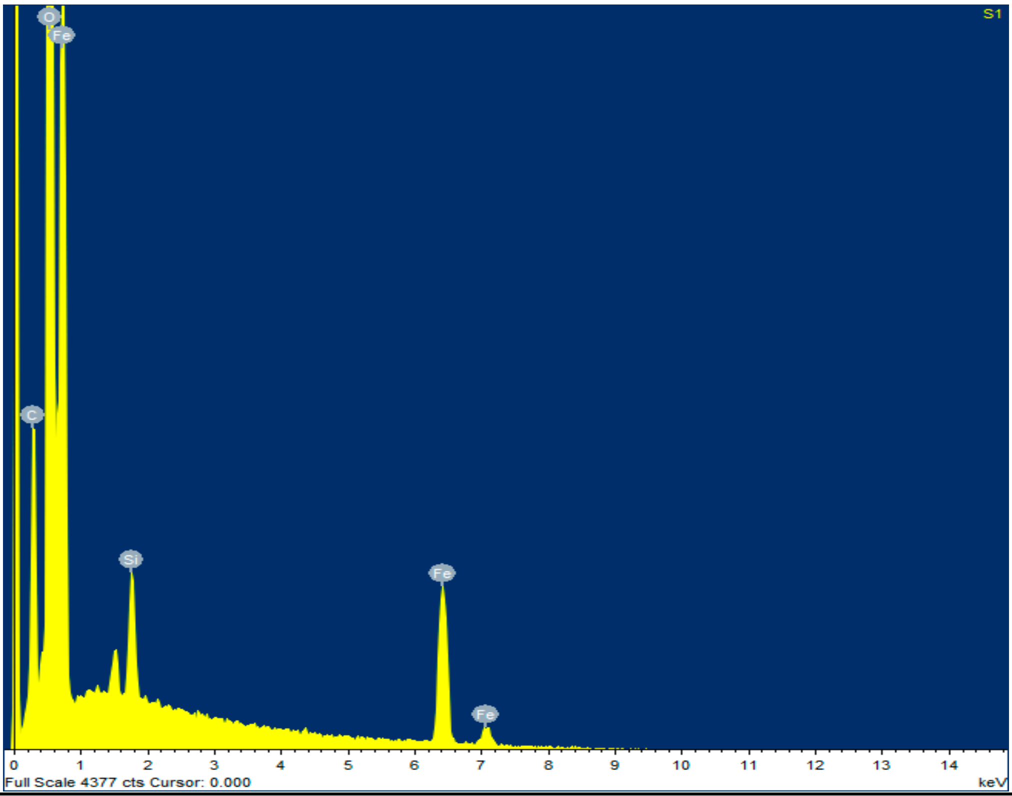

Element	Weight %	Atomic %
C K	7.19	16.43
O K	30.46	52.27
Si K	1.33	1.30
Fe L	61.03	30.00
Totals	100.00	100.00

Figure S1. EDX elemental analysis of APTS-coated magnetic nanoparticles.

(A)

(B)

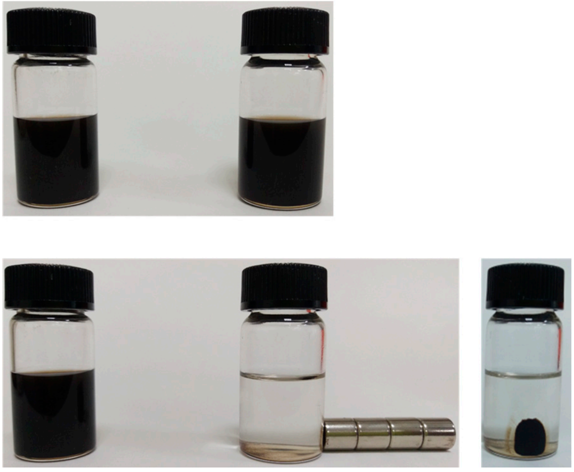

Figure S2. The synthesized magnetic nanoparticles were attracted by a magnet, indicating its magnetic characteristics. (**A**) Magnetic nanoparticles distributed in suspension (**B**) magnetic nanoparticles could be very easily and efficiently collected from the suspension using a magnet.
